# The Localisation of the Mental Faculties

**Published:** 1902-04-26

**Authors:** 


### THE LOCALISATION OF THE MENTAL FACULTIES.

Those who, with Hughlings Jackson, believe that there are "higher centres" in the brain which form the substrata for the higher mental operations, have all, like him, located these centres in the frontal lobes; and as the posterior portion of the frontal convolutions has been demonstrated to control special motor functions, mental control, if resident at all in these lobes, must, says Dr. Phelps, be located in their prefrontal region. Dr. Phelps, however, goes further and maintains not only that such control probably resides in the prefrontal region, but that it is located in the left to the exclusion of the right lobe. This he pointed out in a series of cases published in the *New York Medical Journal* in 1894-5, and he now publishes [1] some more examples of injury and disease showing the same thing. From an analysis of the whole series he makes three generalisations. (1) In every instance but two in which consciousness was retained or regained and the mental faculties were not perverted by general delirium, laceration involving the left frontal lobe was attended by default of intellectual control, and the lesion was usually, if not always, of the prefrontal region and implicated either its superior or inferior surface. Subcortical disintegration, or deep or extensive laceration of the cortex, was specially characterised by abrogation of mental power, and superficial laceration by aberration in its manifestations. (2.) In every instance in which the laceration was confined to the right lobe the mental faculties were unaffected, except as they were obscured by stupor or delirium occasioned by coincident general lesion. (3.) Compression or contusion of the left lobe only exceptionally produced specific intellectual disturbance.

This generalisation is, he says, based upon an examination of an entire series of 295 cases, in which the history was supplemented by necropsy.

[1] Amer. Jour. Med. Sc., April 1902.

